# Weight Change and Associated Factors in Long-Term Breast Cancer Survivors

**DOI:** 10.1371/journal.pone.0159098

**Published:** 2016-07-08

**Authors:** Hye-Yeon Koo, Young-Gyun Seo, Mi-Hee Cho, Min-Jung Kim, Ho-Chun Choi

**Affiliations:** 1 Department of Family Medicine, Seoul National University Hospital, Seoul, Korea; 2 Department of Family Medicine, Healthcare System Gangnam Center, Seoul National University Hospital, Seoul, Korea; University of North Carolina School of Medicine, UNITED STATES

## Abstract

**Purpose:**

Weight gain often occurs after breast cancer diagnosis and significantly impacts the general health of cancer survivors. While the number of breast cancer survivors is increasing, few studies have reported data on weight change beyond 5 years post-diagnosis. We investigated weight change and associated factors in long-term survivors of breast cancer.

**Patients and Methods:**

Medical records were reviewed on 1363 breast cancer patients and a total of 822 women who had survived beyond 5 years since diagnosis were included in the final analysis. The association between demographic, anthropometric, lifestyle, cancer related factors (including time since diagnosis, treatment modality, pathologic stage, and hormone receptor status), and weight-change over 5 years were examined.

**Results:**

During an average 8.2 years of follow-up time, mean weight gain was 0.32kg (p = 0.017). 175 (21.3%) patients had gained more than 5% of their weight at diagnosis and their average gain was 5.55kg. Body mass index (BMI) at diagnosis, age at diagnosis, aromatase inhibitor (AI) use, heavy drinking, and type of surgery were associated with relative weight gain (≥5%) in univariate analysis (all p-values<0.05). Patients who were non-obese at diagnosis showed weight gain, while those who were obese at diagnosis lost weight (0.78kg,−1.11kg, respectively, p<0.001). In multivariate analysis, the non-obese group showed odds ratio of 2.7 (p = 0.001) relative to the obese group. Younger age group (age 18–54 years) showed odds ratio of 1.9 (p = 0.021) relative to the older age group (age 55–75 years), and patients who did not use AI showed odds ratio of 2.2 (p = 0.006) relative to women who did.

**Conclusion:**

Long-term breast cancer survivors who were non-obese at diagnosis are more likely to gain weight than obese survivors. Younger survivors and survivors who have never used AI are also likely to gain weight.

## Introduction

There is considerable evidence that weight gain often occurs after a patient receives a diagnosis of breast cancer. Numerous studies have reported that most of the breast cancer survivors experience weight gain, gaining on average 1.0–6.0kg [1–9]. Suggested mechanisms for post-diagnosis weight gain include decreased physical activity and chemotherapy-associated metabolic changes [1, 5, 9].

A number of investigations have been carried out to identify predictors of weight gain after breast cancer diagnosis. Chemotherapy has been found to be positively associated with weight gain in most studies [2, 5, 10–13], but not in all [14–15]. Several studies reported that premenopausal women are more likely to gain weight [2, 11–13], while others have found no such association [10, 14–16]. Other factors postulated to be associated with weight gain, albeit inconsistently, include younger age and lower body mass index at diagnosis [6, 7, 10–12, 17].

Post-diagnosis weight gain has received attention for its adverse effect on recurrence rate and survival time observed in some earlier studies [11, 17–21] but not in others [7, 8, 12, 22]. Its influence on cancer-specific prognosis remains unclear [1, 9]. However, it is evident that weight gain has a significant impact on the overall health of breast cancer survivors [1, 3, 23]. Weight gain, and consequent obesity, after breast cancer diagnosis might increase the risk of chronic disease [24–26], and has been associated with increased risk of all-cause mortality and cardiovascular disease mortality [21]. These findings suggest that weight gain might even affect the long-term health (including comorbidity or non-cancer death) of breast cancer survivors who have been cured of the cancer itself.

The number of long-term breast cancer survivors is increasing [27, 28]. To develop general health management strategies for this population, it is important to determine whether weight gain persists in long-term survivors and to characterize possible correlates of weight gain. Few studies have reported data on weight change beyond five years post-diagnosis and even they are limited to weight change within six years [13, 29, 30]. Considering that a number of women survive for more than five years, investigations of long-term patterns of weight change are needed. Consequently, we examined weight changes beyond five years post-diagnosis of breast cancer. We considered factors related to cancer itself, its treatment, and life style factors.

## Patients and Methods

### Study population

A retrospective chart review was performed on all women with breast cancer who visited the breast cancer survivor clinic in the Cancer Health Promotion Center at Seoul National University Hospital, between March 2011 and March 2015. This clinic mainly takes responsibility for long-term survivorship care of cancer survivors who were referred from surgeons or medical oncologists. Patients were included if they met all of the following criteria: (1) histologically confirmed and operable stage 0–IIIA breast cancer, (2) diagnosed with breast cancer more than five years before clinic visit, (3) had received surgical treatment for cancer (total mastectomy or breast-conserving surgery), and (4) 18–75 years old at time of diagnosis. Patients were excluded if they met any of the following criteria: (1) history of other cancers, (2) evidence of distant metastases, (3) primary surgical treatment for breast cancer received elsewhere, (4) lack of body weight records at either the time of diagnosis or clinic visit. A total of 1,363 breast cancer patients who visited the breast cancer survivor clinic were identified between March 2011 and March 2015. Of these, 822 met the eligibility criteria. This study was approved by the Institutional Review Board at the Seoul National University Hospital. Since it was a retrospective chart review study, the IRB waived the requirement for informed consent. (IRB No: H-1507-130-689)

### Anthropometric measurements

Data on body weight and height at diagnosis were obtained from recorded baseline measurements prior to surgery. Weight at first visit to the cancer survivor clinic after surgery was measured by medical professionals. Patients were weighed on the same scale in light clothing and without shoes, and the measurement was recorded to the nearest 0.1kg. Body mass index (BMI) was categorized as normal weight (BMI<23kg/m^2^), overweight (BMI 23–25kg/m^2^), or obese (BMI≥25kg/m^2^) according to the World Health Organization criteria for Asians [31]. Relative percent weight changes between diagnosis and clinic visit after surgery were calculated by ((weight at clinic visit—weight at diagnosis)/ weight at diagnosis) ×100. Patients were then categorized into three groups: gain (≥5% gain), stable (-5 to 5% change), or loss (≥5% loss).

### Demographic, clinical, and lifestyle characteristics

Information on demographic and clinical characteristics was obtained through medical record review. Data included age at diagnosis, time between diagnosis and clinic visit, type of surgery, history of adjuvant treatment (chemotherapy, radiotherapy, any hormone therapy, aromatase inhibitor use, and tamoxifen use), pathologic stage, histologic type of tumor, and hormone receptor status. Pathologic stage was calculated according to the American Joint Committee on Cancer (6th Edition of the Cancer Staging Manual).

Data on lifestyle characteristics were obtained from medical records, recorded by the attending physician at the clinic visit. Patients were asked about the type, duration, and frequency of usual physical activities for the month prior to the clinic visit. Intensity of each activity was assessed using the classification made by Ainsworth et al [32]. Physical activity level was dichotomized as “sufficient” (≥150min/week of moderate-intensity activity or ≥75min/week of vigorous-intensity activity) or “insufficient” (<150min/week of moderate-intensity activity or<75min/week of vigorous-intensity activity) according to the American College of Sports Medicine and American Heart Association’s recommendations (2007). Smoking status was categorized as “no” (never a smoker) or “yes” (a past or current smoker). Drinking was defined as having drunk any alcohol more than three drinks per day or more than seven drinks per week according to the National Institute on Alcohol Abuse and Alcoholism’s definition of heavy drinking (2000).

### Data analysis

Absolute weight change from cancer diagnosis to clinic visit was calculated. The significance of weight changes over time was examined using the paired t-test.

The associations between demographic, anthropometric, lifestyle, cancer-related variables, and weight change were then examined. The t-test was conducted to compare differences in weight change across categorical variables. Spearman’s rank correlation analysis was performed to investigate the relationship between weight change and continuous variables. The associations between relative weight gain (≥5%) and demographic, anthropometric, lifestyle, and cancer-related variables were also examined. Two-way tables and chi-squared or Fisher’s exact tests were used to compare proportions of category of weight gain by other categorical variables. Simple logistic regression analysis was conducted to examine the association between weight gain and continuous variables.

Multivariate logistic regression analysis was performed with relative weight gain as the dependent variable. Variables associated with weight gain at a level of p<0.05 in the univariate analysis were considered for inclusion in the final multivariate model. Multicollinearity was examined using the variance inflation factor. All analyses were conducted using STATA MP statistical software, version 14.0 (College Station, TX). All tests were two-sided and the significance levels were set at p < 0.05.

## Results

### Description of the study population

The demographic, anthropometric, lifestyle and cancer-related characteristics of the study population are summarized in Table 1. Mean patient age at diagnosis was 49.4 years. Mean patient body weight was 57.8 (±8.0) kg, and mean BMI was 23.3 (±3.1) kg/m^2^ at the time of diagnosis. At diagnosis, 199 (24.2%) patients were categorized as obese, 196 (23.8%) were overweight, and 427 (52.0%) were of normal weight. Only 108 (13.1%) patients met the criteria for sufficient levels of physical activity, and 571 (69.5%) had insufficient physical activity. The majority of women had no history of smoking; only 8 women (1.0%) were past or current smokers. Most women reported drinking no alcohol or low-risk drinking; 42 (5.2%) reported heavy drinking. In terms of cancer-related characteristics, the mean interval from diagnosis to clinic visit was 8.2 years. In terms of intervention, 468 (56.9%) patients received breast conserving surgery, 354 (43.1%) received mastectomy, 442 (53.8%) received chemotherapy, 464 (56.5%) received radiotherapy, and 519 (63.1%) received hormone therapy.

**Table 1:** Characteristics of the Study Population (n = 822).

	**Mean±SD or Number (%)**
**Age at diagnosis (years)**	49.4y±8.6
18–44	261 (31.8)
45–54	354 (43.1)
55–75	207 (25.2)
**Height (cm)**	157.7 ±5.0
**Weight at diagnosis (kg)**	57.8 ±8.0
**BMI at diagnosis (kg/m**^**2**^)	23.3 ±3.1
**Obesity at diagnosis**	
Normal (BMI<23kg/m^2^)	427 (52.0)
Overweight (BMI 23–25kg/m^2^)	196 (23.8)
Obese (BMI ≥25kg/m^2^)	199 (24.2)
**Smoking** ^a^	
Yes	8 (1.0)
No	680 (82.7)
**Drinking** ^b^	
Yes	42 (5.1)
No	645 (78.5)
**Physical activity** ^c^	
Insufficient	571 (69.5)
Sufficient	108 (13.1)
**Time since diagnosis (years)**	8.2y±2.6
5–10	571 (69.5)
10–15	251 (30.5)
**Type of surgery**	
Breast conserving surgery	468 (56.9)
Mastectomy	354 (43.1)
**Chemotherapy**	
Yes	442 (53.8)
No	380 (46.2)
**Radiotherapy**	
Yes	464 (56.5)
No	358 (43.6)
**Hormone therapy** ^d^	
Yes	519 (63.1)
No	303 (36.9)
**Aromatase inhibitor use**	
Yes	192 (23.4)
No	630 (76.6)
**Tamoxifen use**	
Yes	361 (43.9)
No	461 (56.1)
**Pathologic stage**	
0	75 (9.1)
I	355 (43.2)
II	350 (42.6)
IIIA	42 (5.1)
**Histologic type**	
Infiltrating ductal carcinoma or DCIS	761 (92.6)
Infiltrating lobular carcinoma or LCIS	13 (1.6)
Others	48 (5.8)
**Estrogen receptor** ^e^	
Positive	502 (61.1)
Negative	308 (37.5)
**Progesterone receptor** ^f^	
Positive	416 (50.6)
Negative	393 (47.8)

Abbreviations: SD, standard deviation; BMI, body mass index;; DCIS, ductal carcinoma in situ; LCIS, lobular carcinoma in situ.

^**a**^ Data were missing on smoking status in 134 (16.3%) patients.

^**b**^ Data were missing on drinking status in 135 (16.4%) patients.

^**c**^ Data were missing on physical activity level in 143 (17.4%) patients.

^**d**^ Hormone therapy: any hormone therapy including aromatase inhibitor, tamoxifen, gorsereline, farestone, etc.

^**e**^ Data were missing on estrogen receptor status in 12 (1.5%) patients.

^**f**^ Data were missing on progesterone receptor status in 13 (1.6%) patient.

### Weight changes

Weight changes from cancer diagnosis to clinic visit are summarized in Table 2. The overall mean weight change was 0.32kg (95% confidence interval (CI): 0.06–0.59, p = 0.017). Patients who visited the clinic 5–10 years post-diagnosis (n = 571) had a mean weight gain of 0.4kg (95% CI: 0.09–0.71, p = 0.013), while patients who visited the clinic 10–15 years post-diagnosis (n = 251) had a mean gain of 0.16kg (95% CI: -0.35–0.68, p = 0.536). A total of 175 (21.3%) patients had gained more than 5% of their weight at diagnosis, and their average weight gain was 5.55kg. The number of patients who lost more than 5% of weight was 135 (16.4%), fewer than the number of patients who gained weight. The number of overweight or obese patients increased from 395 (48.1%) at diagnosis to 420 (51.1%) at clinic visit.

**Table 2:** Weight Change Trends According to Time since Diagnosis.

	**Time since diagnosis**		
	**Overall (n = 822)**	**5–10yr (n = 571)**	**10–15yr (n = 251)**
	**Mean±SD or Number (%)**	**Mean±SD or Number (%)**	**Mean±SD or Number (%)**
**Absolute weight change (kg)**	0.32±3.89	0.40±3.78	0.16±4.13
**BMI change (kg/m**^**2**^)	0.12±1.56	0.15±1.51	0.05±1.66
**Weight change classification**			
Loss (≤-5%)	135 (16.4)	87 (15.2)	48 (19.1)
Stable (-5~5%)	512 (62.3)	361 (63.2)	151 (60.2)
Gain (≥5%)	175 (21.3)	123 (21.5)	52 (20.7)

Abbreviations: SD, standard deviation; BMI, body mass index

### Predictors of weight change

Absolute weight change from diagnosis to clinic visit was significantly correlated with weight and BMI at diagnosis (r = -0.177, p<0.001 and r = -0.230, p<0.001, respectively). As can be seen in Table 3, patients who were of normal weight or overweight at diagnosis (n = 623) showed weight gain, while those who were obese at diagnosis (n = 199) lost weight after cancer treatment (0.78kg and -1.11kg, respectively, p<0.001). Among 175 patients who gained more than 5%, most (69.1%) were of normal weight at the time of diagnosis. When comparing the odds ratio for relative weight gain (≥5%), the non-obese group showed higher odds ratio (OR 2.6, p<0.001) relative to the obese group. Age at diagnosis also showed a negative correlation with weight change (r = -0.169, p<0.001). When comparing the relative weight gain among age groups, the younger (18–54 years) age group showed higher odds ratio for weight gain (OR 2.5, p<0.001) relative to the older age group (age 55–75 years). Heavy drinking was found to be associated with relative weight gain (OR 2.1, p = 0.025). Smoking and physical activity were not associated with weight changes (Table 3).

**Table 3:** General Characteristics and Weight Change after Breast Cancer Diagnosis.

	**Absolute weight change (kg)**			**Relative weight gain (≥5%)**		
	**Mean**	**SD**	**p-value**	**Number (%)**	**OR (95% CI)**	**p-value**
**Age at diagnosis (years)**			0.011			<0.001
18–54	0.53	3.94		151 (24.6)	2.5 (1.6–3.9)	
55–75	-0.27	3.71		24 (11.6)	1	
**BMI at diagnosis (kg/m**^**2**^)			<0.001			<0.001
<25 (normal or overweight)	0.78	3.74		153 (24.6)	2.6 (1.6–4.2)	
≥25 (obese)	-1.11	4.01		22 (11.1)	1	
**Smoking**			0.506			0.279
Yes	1.24	2.91		3 (37.5)	2.2 (0.5–9.2)	
No	0.32	3.92		147 (21.6)	1	
**Drinking**			0.189			0.025
Yes	1.10	4.07		15 (35.7)	2.1 (1.1–4.1)	
No	0.28	3.90		135 (20.9)	1	
**Physical activity**			0.375			0.494
Insufficient	0.39	3.97		128 (22.4)	1.2 (0.7–2.0)	
Sufficient	0.02	3.59		21 (19.4)	1	

Abbreviations: SD, standard deviation; BMI, body mass index.

Data were calculated from unadjusted analyses.

Table 4 presents the univariate analyses of associations between weight changes and cancer-related factors. Patients who received breast conserving surgery gained weight, while those who received mastectomy lost a small amount of weight (0.64kg and -0.09kg, respectively, p = 0.007). Patients who did not use aromatase inhibitors showed weight gain, whereas those who did showed weight loss (0.58kg and -0.52kg, respectively, p<0.001). Time since diagnosis, chemotherapy, radiotherapy, hormone therapy, tamoxifen use, pathologic stage, histologic type of cancer, and hormone receptor status were not associated with weight changes.

**Table 4:** Cancer-related Factors and Weight Change after Breast Cancer Diagnosis.

	**Absolute weight change (kg)**			**Relative weight gain (≥5%)**		
	**Mean**	**SD**	**p-value**	**Number (%)**	**OR (95% CI)**	**p-value**
**Time since diagnosis(years)**			0.426			0.790
5–10	0.40	3.78		123 (21.2)	1.1 (0.7–1.5)	
10–15	0.16	4.13		52 (20.5)	1	
**Type of surgery**			0.007			0.049
Breast conserving surgery	0.64	3.72		111 (23.7)	1.4 (1.0–2.0)	
Mastectomy	-0.09	3.99		64 (18.1)	1	
**Chemotherapy**			0.311			0.986
Yes	0.45	3.87		94 (21.3)	1.0 (0.7–1.4)	
No	0.18	3.92		81 (21.3)	1	
**Radiotherapy**			0.453			0.286
Yes	0.41	3.91		105 (22.6)	1.2 (0.9–1.7)	
No	0.21	3.88		70 (19.6)	1	
**Hormone therapy**			0.231			0.929
Yes	0.20	4.13		111 (21.4)	1.0 (0.7–1.4)	
No	0.54	3.44		64 (21.1)	1	
**Aromatase inhibitor use**			<0.001			<0.001
Yes	-0.52	3.88		23 (12.0)	1	
No	0.58	3.86		152 (24.1)	2.3 (1.5–3.7)	
**Tamoxifen use**			0.877			0.221
Yes	0.35	4.04		84 (23.3)	1.2 (0.9–1.7)	
No	0.31	3.78		91 (19.7)	1	
**Pathologic stage**			0.218			0.271
0, I	0.48	3.70		98 (22.8)	1.2 (0.9–1.7)	
II, IIIA	0.15	4.09		77 (19.6)	1	
**Histologic type**			0.160			0.106
IDC or DCIS	0.27	3.89		157 (20.6)	0.6 (0.3–1.1)	
Other histologic types ^a^	1.00	3.94		18 (29.5)	1	
**Estrogen receptor**			0.423			0.671
Yes	0.20	3.92		109 (21.7)	1.1 (0.8–1.5)	
No	0.46	3.84		63 (20.5)	1	
**Progesterone receptor**			0.700			0.260
Yes	0.27	3.91		95 (22.8)	1.2 (0.9–1.7)	
No	0.37	3.87		77 (19.6)	1	

Abbreviations: SD, standard deviation; IDC, Infiltrating ductal carcinoma; DCIS, ductal carcinoma in situ

Data were calculated from unadjusted analyses.

^a^ Other histologic types: Any histologic types other than infiltrating ductal carcinoma or ductal carcinoma in situ, including infiltrating lobular carcinoma, lobular carcinoma in situ, etc.

Table 5 shows the final model for weight gain after breast cancer diagnosis. Multivariate logistic regression analysis was performed including potential predictor variables that were associated with relative weight gain in univariate analyses. Before entering the five factors into a multivariate model, multicollinearity was checked. No significant multicollinearity was found using the variance inflation factor (range: 1.02–1.13) among the five variables. In the final model, BMI at diagnosis, age at diagnosis, and aromatase inhibitor use were found to be significantly associated with weight gain (p-values for each factors were 0.001, 0.021, and 0.006 respectively).

**Table 5:** Factors Related to Relative Weight Gain (≥5%) in Long-term Breast Cancer Survivors.

**Variables**	**aOR**	**95% CI**	**p-value**
**BMI <25 kg/m2 at diagnosis (ref. BMI ≥25.0 kg/m2)**	2.7	1.5–4.8	0.001
**Age <55 years at diagnosis (ref. ≥55 years)**	1.9	1.1–3.3	0.021
**No AI use (ref. AI use)**	2.2	1.3–4.0	0.006
**Drinking >3 drinks/day or >7 drinks/week (ref.≤3 drinks/day&≤7 drinks/week)**	1.8	0.9–3.5	0.101
**Breast conserving surgery (ref: mastectomy)**	1.3	0.9–1.9	0.182

Abbreviations: aOR, adjusted odds ratio; CI, confidence interval; ref., reference group; BMI, body mass index; AI, aromatase inhibitor.

Data were calculated from multivariate logistic regression analysis after adjustment for related variables.

## Discussion

In this study of long-term survivors of breast cancer, only a small amount of weight gain was observed over 5 years post-diagnosis. The average weight gain was 0.32kg, which is far less than gains observed within 5 years post-diagnosis in most studies [1, 7, 10, 31]. However, this result needs to be interpreted with caution because ethnic differences might exist in weight gain. Previous research has suggested that there are differences in patterns of weight change between Korean and Western breast cancer survivors [14, 15, 33]. In a study by Jeon et al., Korean breast cancer patients gained an average of 3.4kg during the adjuvant TAC (docetaxel-doxorubicin-cyclophosphamide) chemotherapy period, but this gain was not sustained at 12 or 24 months post-surgery (mean weight change: -0.19kg and -0.37kg, respectively) [33]. This finding implies that weight gain may only be transient in Korean breast cancer survivors. However, previous studies were of small sample sizes and limited to patients who received chemotherapy.

Studies conducted in the West demonstrated that weight gain persisted for 2–6 years after cancer treatment [6, 7, 29, 30]. In a prospective study of Australian women with early breast cancer [29], progressive weight gain was observed over 72months of follow-up. While overall median weight gain of participants was small (0.7kg), 80% of women who gained weight experienced a larger gain than age-matched norms. The proportion of overweight or obese women increased from 57–68% between 6 and 72 months after surgery. In the Women’s Healthy Eating and Living (WHEL) study, which examined weight changes of 2,972 breast cancer survivors, mean body weight progressively increased for two years post-diagnosis, then plateaued [30]. Only 10% of participants returned to their pre-diagnosis weight during 6 years of follow-up. These findings suggest that weight gain after breast cancer diagnosis may not be transitory but may persistent, implying the need for further investigation into long-term weight change patterns in Asian and Western women.

In the present study, patients who were of normal weight at breast cancer diagnosis showed an increase in weight after at least five years post-surgery, while obese patients experienced a weight loss. Of the 175 women who gained more than 5% of baseline weight, 69% were of normal weight at diagnosis. A negative relationship was identified between BMI at diagnosis and the risk for weight gain. This finding is consistent with previous studies that have reported weight changes over a relatively short period of time [7, 10, 26]. For example, a observational study of breast cancer patients found that women who were of normal weight(BMI<25kg/m^2^) at diagnosis gained an average of 2kg during the following year, while overweight and obese women lost 1.4kg and 1.9kg, respectively [26]. In contrast, in the general population women with higher baseline BMI are known to gain more weight [34, 35]. These contrasting trends between women with breast cancer and cancer-free women might have been caused by cancer survivor-specific mechanism of weight change.

The exact reason has not been identified to date, but the fact that similar weight change patterns were observed in both short- and long-term survivors implies that a persistent factor affecting weight gain after diagnosis might exist. One possible explanation is that a fact that obesity has a negative impact on cancer prognosis and general health, thus obese women may have become more conscious of their weight after the diagnosis of cancer. This could have led to better health-related behaviors compared with normal weight women [26]. Several studies have suggested that the stress of receiving a cancer diagnosis and undergoing treatment might have a persistent negative impact on behavioral factors, such as diet and physical activity, which are associated with sustained weight gain [1, 6, 10]. Our study failed to find a significant association between physical activity level and weight change; however, this result must be interpreted with caution because information on physical activity at diagnosis was not collected. It is possible that a reduction in physical activity between the time of diagnosis and clinic visit was missed.

Weight gain is known to increase the risk of coronary heart disease and premature death even if it is in the range of normal BMI [36, 37]. If weight gain in women with normal weight is not recovered quickly and patients become overweight or obese, the risk of associated comorbidities may increase further. A number of investigations on weight management strategies for breast cancer survivors have been conducted recently [38, 39]. Considering the finding of the current study, weight control interventions may have to be sustained over a long-term period, especially in women who were of normal weight at diagnosis.

Factors other than BMI associated with weight gain in the present study were age at diagnosis and aromatase inhibitor use. Age at diagnosis showed a negative correlation with weight change, consistent with earlier studies [6, 7, 10]. The exact mechanism is not clear, but similar patterns of weight gain have been observed in the general population of women [40, 41]: a report on body weight and weight changes in U.S. adults [40] showed that adults younger than 55 years tend to gain weight while those 55 years and older tend to lose weight when followed up for 10 years. Age might be a factor affecting weight change in all adults, including cancer survivors and the healthy population, although the magnitude of weight change can differ [2, 10]. In our study, patients who had never used aromatase inhibitors were more likely to gain weight than those who had in our study; however, most previous investigations found no significant association [9, 15, 26]. Since aromatase inhibitors are usually used in postmenopausal women, the association observed in our study might have been induced by the effect of menopausal status. It is likely that women who had used aromatase inhibitors were already postmenopausal at the time of diagnosis, while many of the women who had not used aromatase inhibitor were premenopausal at diagnosis and experienced menopause during cancer treatment. Several studies have suggested that treatment-induced menopause maybe associated with weight gain by accelerating menopause-related physiologic changes such as fat accumulation [1, 2, 42]. However, the association remains controversial [10, 14–16], and further research should include data on changes in menopausal status. History of chemotherapy, which was associated with weigh gain in most reports, did not show a significant association in the current study: this result is in line with other studies conducted in Korea [14–15].

There are some limitations in this study. It was a retrospective, cross-sectional study. Participants were recruited from a single university hospital, so they may not be representative of the entire population of breast cancer survivors in Korea; however, this clinic takes responsibility for about 2–3% of Korean long-term breast cancer survivors [43–44]. Because of limitations in data collected by history taking at the clinic, physical activity level was not quantified by a standard instrument such as the International Physical Activity Questionnaire. In addition, data on other factors related to energy balance, such as dietary intake, was not collected. Strengths of this study include being the only study to specifically examine weight changes in long-term breast cancer survivors who have survived beyond 5 years post-diagnosis. Few data exist on long-term weight change patterns of women with breast cancer. In addition, this study has a relatively large sample size and included data on various clinical characteristics that are potential risk factors for weight gain, including cancer-related factors.

## Conclusion

This is the first study to investigate weight change after breast cancer diagnosis in women who have survived beyond 5 years. Only small weight gains were observed during an average 8.2years of follow up in Korean breast cancer survivors, implying that weight gain after breast cancer diagnosis may differ among ethnic groups and time periods. However, survivors who were non-obese at diagnosis were more likely to gain weight than obese survivors. Younger survivors and survivors who had never used aromatase inhibitors were also likely to experience weight gain. Further studies should examine long-term weight change patterns of Asian and Western breast cancer survivors, and elucidate the relationship between weight gain and general health in this population.
